# An adaptive randomised placebo controlled phase II trial of antivirals for COVID-19 infection (VIRCO): A structured summary of a study protocol for a randomised controlled trial

**DOI:** 10.1186/s13063-020-04766-5

**Published:** 2020-10-13

**Authors:** James H. McMahon, Jillian S. Y. Lau, Janine Roney, Benjamin A. Rogers, Jason Trubiano, Joseph Sasadeusz, James S. Molton, Bradley Gardiner, Sue J. Lee, Jennifer F. Hoy, Allen Cheng, Anton Y. Peleg

**Affiliations:** 1 Department of Infectious Diseases, Alfred Hospital and Monash University, Melbourne, Australia; 2 Department of Infectious Diseases, Monash Medical Centre, Melbourne, Australia; 3 Department of Infectious Diseases, Eastern Health, Melbourne, Australia; 4 Department of Infectious Diseases, Austin Hospital, Melbourne, Australia; 5 Department of Infectious Diseases, Royal Melbourne Hospital, Melbourne, Australia; 6 Department of Infectious Diseases, Western Health, Melbourne, Australia; 7 Epworth Healthcare, Melbourne, Australia

**Keywords:** COVID-19, Randomised controlled trial, protocol, favipiravir, treatment, community, adaptive

## Abstract

**Objectives:**

**Primary objective:** To determine the efficacy of a candidate antiviral on time to virological cure compared to standard of care within 14 days of randomisation

**Secondary objectives:**

• To determine the safety of the antiviral

• To determine the clinical benefit of the antiviral over placebo according to the WHO 7-point ordinal scale

• To determine the clinical benefit of the antiviral over placebo on time to resolution of clinical symptoms

• To determine the effect of the antiviral over placebo on biomarkers of inflammation and immune activation

**Trial design:**

This is a multi-centre, triple-blind, randomised placebo controlled phase II, 2-arm trial with parallel-group design with allocation ratio 1:1.

**Participants:**

Inclusion Criteria:

• Provision of informed consent by the participant

• Age ≥18 years

• Confirmed SARS-CoV-2 by nucleic acid testing in the past 5 days

• COVID-19 related symptom initiation within 5 days

• Female patients of childbearing potential must have a negative pregnancy test at Screening. Female patients of childbearing potential and fertile male patients who are sexually active with a female of childbearing potential must use highly effective methods of contraception throughout the study and for 1 week following the last dose of study treatment.

Exclusion criteria:

• Known allergy to the study medication

• Is on another clinical trial investigating an antiviral treatment for COVID-19

• Pregnancy

• Patients with severe hepatic dysfunction equivalent to Grade C in the Child-Pugh classification

• Patients with renal impairment requiring dialysis

• Is deemed by the Investigator to be ineligible for any reason

Participants will be recruited from, and the study visits will take place at Alfred Hospital, Monash Health, Austin Health in Victoria, Australia for hospitalised participants as well as recruitment in the community in participants homes for eligible people not requiring hospitalisation.

**Intervention and comparator:**

The first candidate antiviral is favipiravir

Arm 1: Favipiravir 1800 mg favipiravir BD on Day 1 followed by 800 mg BD favipiravir for the next 13 days.

Arm 2: Placebo

**Main outcomes:**

Primary outcome: Time to virological cure as defined by 2 successive throat (or combined nose/throat) swabs negative for SARS-CoV-2 by nucleic acid testing during the 14 days after enrolment.

**Randomisation:**

Randomisation performed at the Alfred Hospital Clinical Trials Pharmacy using computer generated block-randomisation lists with 6 participants per block. Within each block half of the participants will be randomised to the candidate antiviral and the other half to placebo. Randomisation is stratified by study site, with participants enrolled in the community considered as a study site.

**Blinding (masking):**

Study participants, study investigators and the study statistician will be blinded to treatment allocation.

**Numbers to be randomised (sample size):**

The study aims to recruit 190 people (95/arm) with the first candidate antiviral favipiravir

**Trial Status:**

Protocol version 2.0 Dated 31-Jul-2020. Recruitment will take place between July 2020 and December 2020.

**Trial registration:**

clinicaltrials.gov NCT04445467

First posted 24-Jun-2020

**Full protocol:**

The full protocol is attached as an additional file, accessible from the Trials website (Additional file 1). In the interest in expediting dissemination of this material, the familiar formatting has been eliminated; this Letter serves as a summary of the key elements of the full protocol.

## Supplementary information

**Additional file 1.** Full Study Protocol.

## Data Availability

Not applicable.

