# Supplementary material for: An adaptive randomised placebo controlled phase II trial of antivirals for COVID-19 infection (VIRCO): A structured summary of a study protocol for a randomised controlled trial
Source: Trials. 2020 Oct 13;21:847. doi: 10.1186/s13063-020-04766-5 (PMC7552590; doi:10.1186/s13063-020-04766-5)
Supplement: Supplementary file 1 — Additional file 1. Full Study Protocol. [file 13063_2020_4766_MOESM1_ESM.pdf]

An adaptive randomised placebo controlled phase II trial of antivirals for COVID-19 infection (VIRCO)

Protocol Number: 66223
Version: # 2.0
Date: July 31st , 2020

Author:
Dr James McMahon

Sponsor:
Alfred Health

CONFIDENTIAL
This document is confidential and the property of Alfred Health. No part of it may be transmitted, reproduced, published, or used without prior written authorization from the institution.

Statement of Compliance
This study will be conducted in compliance with all stipulations of this protocol, the conditions of the ethics committee approval, the NHMRC National Statement on ethical Conduct in Human Research (2007 and all updates), the integrated Addendum to ICH E6 (R1): Guideline for Good Clinical Practice E6 (R2) dated 09 November 2016 annotated with TGA comments and the NHMRC guidance Safety monitoring and reporting in clinical trials involving therapeutics goods (EH59, 2016).

Approval:

______________________________	______________
Coordinating Principal Investigator Signature: **_Dr James McMahon_**	*Date*

TABLE OF CONTENTS

PROTOCOL SYNOPSIS **5**

Glossary of Abbreviations and Terms **8**

Investigator Agreement **9**

1 RESPONSIBILITIES AND PARTNERSHIPS **10**

1.1 SPONSOR 10

1.2 PROTOCOL IDENTIFYING NUMBER 10

1.3 DATE OF PROTOCOL 10

1.4 COORDINATING PRINCIPAL INVESTIGATOR 10

1.5 INVESTIGATORS 10

1.6 PROTOCOL STEERING COMMITTEE (PSC) 11

2 INTRODUCTION **11**

2.1 BACKGROUND AND RATIONALE 11

2.2 CANDIDATE ANTIVIRAL 12

3 PROJECT AIM **12**

4 OBJECTIVES **12**

4.1 PRIMARY OBJECTIVE 12

4.2 SECONDARY OBJECTIVES 12

5 OUTCOMES AND OUTCOME MEASURES **13**

5.1 PRIMARY ENDPOINT 13

5.2 SECONDARY ENDPOINTS 13

6 EXPERIMENTAL DESIGN **13**

6.1 STUDY SITE 13

6.2 STUDY DESIGN 14

6.3 DURATION OF STUDY 14

6.4 STUDY IMPLEMENTATION TIMELINE 14

7 ELIGIBILITY CRITERIA **14**

7.1 INCLUSION CRITERIA 14

7.2 EXCLUSION CRITERIA 14

7.3 TREATMENT DISCONTINUATION, PARTICIPANT WITHDRAWALS AND LOSSES TO FOLLOW-UP 15

7.3.1 TREATMENT DISCONTINUATION – PARTICIPANT REMAINS IN TRIAL 15

7.3.2 WITHDRAWAL OF CONSENT – PARTICIPANT WITHDRAWS FROM ALL TRIAL PARTICIPATION 15

7.3.3 LOSSES TO FOLLOW-UP 16

7.3.4 DEFINITION OF INCLUSION AND REPLACEMENT OF PARTICIPANTS 16

7.3.5 TRIAL CLOSURE 16
7.3.6 TEMPORARY HALT OR EARLY TERMINATION OF THE TRIAL 16
7.4 PARTICIPANT CONSENT 17
8 STUDY PROCEDURES 18
8.1 SCHEDULE OF ACTIVITIES 18
8.2 RANDOMISATION 20
8.3 VISIT 1 – SCREENING / BASELINE 20
8.3.1 VISITS 2, 4, 6, AND 8 21
8.3.2 VISITS 3, 5 AND 7 21
8.3.3 VISIT 9 22
8.3.4 VISIT 10 22
8.3.5 VISITS 11, 12 AND 13 (OPTIONAL) 22
9 LABORATORY SAMPLES 23
9.1 BLOOD SAMPLES 23
9.2 COMBINED NOSE / THROAT SWAB 23
9.3 SAMPLE PROCESSING AND STORAGE 24
10 SAFETY ASSESSMENTS 24
10.1 ADVERSE EVENT (AE) 24
10.2 SERIOUS ADVERSE EVENT (SAE) 24
10.3 SUSPECTED UNEXPECTED SERIOUS ADVERSE REACTION (SUSAR) 25
10.4 ASSESSMENT OF ADVERSE EVENTS 25
10.5 RECORDING OF ADVERSE EVENTS 25
10.6 ASSESSING THE RELATEDNESS (CAUSALITY) OF A PARTICIPANT'S AE 27
10.7 REPORTING OF SERIOUS ADVERSE EVENTS 27
10.8 SAFETY MONITORING COMMITTEE 28
10.8.1 INTERIM REVIEW BY SAFETY MONITORING COMMITTEE 28
10.8.2 SMC RECOMMENDATIONS 29
10.9 SPONSOR REPORTING PROCEDURES 29
10.10 RISK MANAGEMENT AND SAFETY 30
10.10.1 CANDIDATE ANTIVIRAL 30
10.10.2 PREGNANCY 30
10.10.3 PHLEBOTOMY 31
10.10.4 COMBINED NOSE / THROAT SWABS 31
11 STATISTICAL ANALYSIS 31
11.1 SAMPLE SIZE CALCULATION 31
11.2 ANALYSIS PLAN 31
11.2.1 ANALYSIS POPULATIONS 31

11.2.2 BASELINE DATA 32
11.2.3 PRIMARY ENDPOINT 32
11.2.4 SECONDARY ENDPOINTS 32
12 ETHICS AND DISSEMINATION 32
12.1 RESEARCH ETHICS APPROVAL & LOCAL GOVERNANCE AUTHORISATION 32
12.2 CONFIDENTIALITY 33
13 DATA COLLECTION, PROCESSING AND STORAGE 33
13.1 SOURCE DATA 33
13.2 DATA CAPTURE METHODS AND STORAGE 33
13.3 RECORD RETENTION 34
13.4 STORAGE OF BIOLOGICAL MATERIAL AND GENETIC TESTING 34
14 PUBLICATION OF STUDY 35
15 REFERENCES 35
16 APPENDICES 37
16.1 APPENDIX A – CANDIDATE ANTIVIRALS 37
16.2 FAVIPIRAVIR 37
16.2.1 DOSING 37
16.2.2 OVERVIEW OF FAVIPIRAVIR 37
16.2.3 STUDY DESIGN 38
16.2.4 DURATION OF STUDY 38
16.2.5 ADDITIONAL INCLUSION CRITERIA 38
16.2.6 ADDITIONAL EXCLUSION CRITERIA 38
16.2.7 ADVERSE EFFECTS 39
16.2.8 TERATOGENIC EFFECTS 40
16.2.9 DRUG-DRUG INTERACTIONS 40
16.2.10 OTHER SAFETY CONSIDERATIONS 41

PROTOCOL SYNOPSIS

<table>
<tr><td>Title</td><td>An adaptive randomised placebo controlled phase II trial of antivirals for COVID-19 infection (VIRCO)</td></tr>
<tr><td>Trial Description</td><td>This is a randomised placebo controlled phase II trial to examine the efficacy of antivirals to treat COVID-19 infection compared to placebo for virological cure and improved clinical outcomes. Individuals will be randomised to the candidate antiviral which in the first instance is Favipiravir or matched placebo. Randomisation will be stratified by study site with participants enrolled in the community considered as a study site. This treatment will be given in addition to the usual standard of care in the participating hospital.

Adaptive design: The interim trial results will be monitored by an independent Safety Monitoring Committee (SMC). The most important task for the SMC will be to assess whether the randomised comparison in the study have provided evidence of an antiviral effect (with a range of uncertainty around the results that is narrow enough) to impact treatment strategies. In such a circumstance, the SMC will inform the Trial Protocol Steering Committee who will amend the trial accordingly. New trial arms can be added as evidence emerges that other candidate therapeutics should be evaluated.</td></tr>
<tr><td>Objectives</td><td>Primary objective
• To determine the efficacy of the antiviral on time to virological cure compared to standard of care

Secondary objectives
• To determine the safety of the antiviral
• To determine the clinical benefit of the antiviral over placebo according to the WHO 7-point ordinal scale
• To determine the clinical benefit of the antiviral over placebo on time to resolution of clinical symptoms
• To determine the effect of the antiviral over placebo on biomarkers of inflammation and immune activation</td></tr>
<tr><td>Outcomes and Outcome Measures</td><td>Primary endpoint
Time to virological cure as defined by 2 successive throat (or combined nose/throat) swabs negative for SARS-CoV-2 by nucleic acid testing

Secondary endpoints
• The frequency of all adverse events definitely, probably or possibly related to study treatment.
• Time from randomisation to an improvement of two points (from the status at randomisation) on the 7-point ordinal scale</td></tr>
</table>

	• Time from randomisation to resolution of clinical symptoms (fever, cough, shortness of breath, cough). Resolution defined as the start of the first 24 hour period when all symptoms are rated as mild or absent and remained this way for 24 hours<br>• Changes in SARS-CoV-2 viral load from nose/throat swabs from baseline over time<br>• Biomarkers taken as part of routine care including total lymphocyte count, CRP, Ferritin and LDH.<br>• Investigational markers of immune response such as markers of immune activation (e.g. IL-2R, IL-6, IL-10, GM-CSF, CXCL10, CCL2, TNF- alpha, HLA-DR+ expression on T-cells) and cellular and humoral (e.g. establishing T-and B-cell memory, development of neutralising antibodies) immune responses
**Trial Population**	Inclusion Criteria<br>• Provision of informed consent by the participant<br>• Age ≥18 years<br>• Confirmed SARS-CoV-2 by nucleic acid testing in the past 5 days<br>• COVID-19 related symptom initiation within 5 days<br>• Female patients of childbearing potential must have a negative pregnancy test at Screening. Female patients of childbearing potential and fertile male patients who are sexually active with a female of childbearing potential must use highly effective methods of contraception throughout the study and for 1 week following the last dose of study treatment.<br><br>Exclusion criteria<br>• Known allergy to the study medication<br>• Is on another clinical trial investigating an antiviral treatment for COVID-19<br>• Pregnancy<br>• Patients with severe hepatic dysfunction equivalent to Grade C in the Child-Pugh classification<br>• Patients with renal impairment requiring dialysis<br>• Is deemed by the Investigator to be ineligible for any reason
**Recruiting Site**	• Alfred Hospital – Victoria, Australia<br>• Monash Health – Victoria, Australia<br>• Other sites (to be confirmed)
**Description of Interventions**	Arm 1: The candidate antiviral – Refer Appendix A- Favipiravir 1800 mg favipiravir BD on Day 1 followed by 800 mg BD favipiravir for the next 13 days.<br>Arm 2: Placebo
**Sample Size**	The sample size calculation for the candidate antiviral Favipiravir was based on estimates from two published studies. In one randomised clinical trial of 236 patients with COVID-19, 61% of people receiving met the primary

	clinical recovery endpoint by day 7, compared to 52% of people receiving Arbidol . (Chen medRxiv doi.org/10.1101/2020.03.17.20037432). In a non-randomised study of 80 people, 90% of individuals receiving Favipiravir reached viral clearance from respiratory tract samples compared to 57% of individuals receiving lopinavir/ritonavir, with a significantly faster rate of clearance in patients who received the selected antiviral candidate (Hazard Ratio (HR) 3.43, 95% CI 1.16 to 10.1) (Cai et al, 2020). Allowing for more conservative estimates, we assumed 80% of patients on Favipiravir and 60% of patients in the placebo arm would reach virological cure by study end, with twice as fast a rate of cure occurring in Favipiravir arm (HR 2.0). Assuming an alpha of 0.05, 86 participants in each arm would allow a log rank comparison with 80% power. Allowing for 10% LFU, this study aims to recruit 190 people (95/arm).
**Trial Duration**	Eligible participants with confirmed COVID-19 infection will be randomised 1:1 to the selected candidate antiviral Favipiravir or placebo in addition to standard of care. Participants are given Favipiravir /Placebo for 14 days. Study participants can be either a hospital inpatient, at home in the community or a combination of inpatient / community. Participants will be followed for a minimum of 28 days. Participants have the option to provide additional blood samples to assess longer term immunological outcomes at additional visits up to 12 months post randomisation
**Participant Duration**	It is expected that participants will be on study for 28 days to assess the primary and secondary outcomes. Visits will be every second day until Day14 then at Days 21 and 28. Visits can occur in the inpatient or community setting depending on where the participant is located. Participants have the option of providing additional blood samples for storage at 3 further visits up to 12 months post randomisation.

Glossary of Abbreviations and Terms

Abbreviation	Description
AE	Adverse Event
ALT	Alanine aminotransferase
COVID-19	Corona Virus Disease 2019
CPI	Coordinating Principal Investigator
CRF/eCRF	Case Report Form/electronic Case Report Form
eGFR	estimated glomerular filtration rate
FBE	Full Blood Examination
GLP	Good Laboratory Practise
HCV	Hepatitis C virus
HIV	Human Immunodeficiency virus
HREC	Human Research Ethics Committee
LFT	Liver function test
PBMC	Peripheral Blood Mononuclear Cell
PK	Pharmacokinetics
qPCR	quantitative Polymerase Chain Reaction
PI	Principal Investigator
PPE	Personal Protective Equipment
RBG	Random Blood Glucose
RGO	Research Governance Office
RNA	Ribonucleic acid
SAE	Serious adverse event
SARS-CoV-2	Severe Acute Respiratory Syndrome Coronavirus 2
SOC	Standard Of Care
SUSAR	Suspected Unexpected Serious Adverse Reaction
TGA	Therapeutics Goods Administration (Australia)
UEC	Urea, Electrolytes and Creatinine
WOCBP	Women of Child Bearing Potential

Site Principal Investigator Agreement

I have read the protocol specified below.

By signing this protocol, I agree to conduct the clinical trial, after approval by a Human Research Ethics Committee or Institutional Review Board (as appropriate), in accordance with the protocol, the principles of the Declaration of Helsinki and the good clinical practice guidelines adopted by the TGA [Integrated Addendum to ICH E6 (R1): Guideline for Good Clinical Practice E6 (R2), dated 9 November 2016 annotated with TGA comments].

Changes to the protocol will only be implemented after written approval is received from the Human Research Ethics Committee or Institutional Review Board (as appropriate), with the exception of medical emergencies.

I will ensure that trial staff fully understand and follow the protocol and evidence of their training is documented on the trial training log.

Protocol Number:

Protocol Title: An adaptive randomised placebo-controlled phase II trial of antivirals for COVID-19 infection (VIRCO)

Protocol Date:

______________________________ ______________

Investigator Signature ***Date***

____

Print Name and Title

Site Name ______________________________

Address ______________________________

____

____

____

Phone Number ______________________________

1 Responsibilities and Partnerships

1.1 Sponsor

Alfred Health, Melbourne, Victoria

1.2 Protocol identifying number

66223
Alfred HREC 406/20
Ethics Research Manager 66223
clinicaltrials.gov NCT04445467

1.3 Date of Protocol

31 July 2020

1.4 Coordinating Principal Investigator

Dr. James H. McMahon, MBBS FRACP MPH PhD
Head Clinical Research Unit
Infectious Diseases Physician, Department of Infectious Diseases,
Alfred Hospital, Melbourne, Australia
Tel: +61 3 9076 6908
Email: james.mcmahon@monash.edu

1.5 Investigators

Professor Allen Cheng, MB BS, FRACP, MPH, MBiostat, PhD
Director, Infection Prevention and Healthcare Epidemiology Unit,
Infectious Diseases Physician, Department of Infectious Diseases,
Alfred Hospital, Melbourne, Australia
Tel: +61 3 9903 0555
Email: allen.cheng@monash.edu

Professor Anton Y. Peleg MBBS FRACP MPH PhD.
Director and Infectious Diseases Physician, Department of Infectious Diseases,
Alfred Hospital, Melbourne, Australia
Tel: +61 3 9076 6900
Email: anton.peleg@monash.edu

Professor Jennifer Hoy, MBBS FRACP Grad.Dip.Epidemiol. & Biostats.
Director, HIV Medicine and Infectious Diseases Physician
Department of Infectious Diseases,
Alfred Hospital, Melbourne, Australia
Tel: +61 3 9076 6900
Email: Jennifer.hoy@monash.edu

Dr Jillian Lau MBBS FRACP

Infectious Diseases Physician, Department of Infectious Diseases,
Alfred Hospital, Melbourne, Australia
Tel: +61 3 9076 6908
Email: jillian.lau@monash.edu

Dr Sue Lee PhD
Biostatistician, Department of Infectious Diseases
Alfred Hospital, Melbourne, Australia
Tel: +61 3 9076 6900
Email: sue.lee@monash.edu

A/Prof. Benjamin A Rogers, MBBS PhD FRACP
Infectious Disease Physician, Monash Health, Clayton. Australia
Tel. +61 3 95944564
Email: ben.rogers@monash.edu

Janine Roney MPH BHSc GradCert (ID) RN
Clinical Research Manager, Department of Infectious Diseases
Alfred Hospital, Melbourne, Australia
Tel: +61 3 9076 2296
Email: j.roney@alfred.org.au

1.6 PROTOCOL STEERING COMMITTEE (PSC)

- Dr James McMahon, Department of Infectious Diseases, Alfred Hospital, Melbourne
- Professor Allen Cheng, Department of Infectious Diseases, Alfred Hospital, Melbourne
- Professor Anton Peleg, Department of Infectious Diseases, Alfred Hospital, Melbourne
- A/Prof. Benjamin A Rogers Infectious Disease Physician, Monash Health, Melbourne. Australia

2 INTRODUCTION

2.1 BACKGROUND AND RATIONALE

In December 2019, a new coronavirus was identified in Hubei province China, that caused a pneumonia syndrome and was fatal in many individuals. This virus named SARS-CoV-2 is the causative agent in the COVID-19 clinical syndrome. The COVID-19 pandemic has infected millions of people in over 150 countries, with approximately 1-2% of individuals infected dying from the infection. This has led to an unprecedented public health and societal response with large demands on the Australian health system. Conducting research in this

climate is critical to try minimise the harm from this infection and protect individuals at risk of becoming infected.

There are currently no medications recommended to treat COVID-19 infection and Australian guidelines say that infected people in Australia only receive treatments in the setting of a clinical trial. Current clinical trials in Australia are large pragmatic open-label studies of hospitalised individuals focusing on two antivirals (Lopinavir/ritonavir and hydroxychloroquine). Many individuals are excluded from these studies because of concern for toxicity and drug interactions from the study drugs. Excluded people include many with: HIV, heart conditions. organ transplants and other immunocompromised people. Therefore, there is a need for studies that can identify whether the drugs are effective at targeting the virus in infected patients and can also adapt to new data from other studies as it becomes available.

Due to the highly infectious nature of COVID-19 and the impact of community based infection on infected people and their contacts there is also a need to study safe treatments in the community that target the virus. This will benefit the infected person and potentially decrease the potential for transmission.

2.2 Candidate Antiviral

The first candidate antiviral for this randomised placebo controlled clinical trial is Favipiravir (Refer Appendices – Candidate Antiviral – Favipiravir)

3 Project Aim

To conduct a clinical trial for people in the hospital and community setting and determine if antivirals to treat COVID-19 infection can clear the virus, are safe and can improve clinical outcomes.

4 Objectives

4.1 Primary Objective

- To determine the efficacy of the antiviral on time to virological cure compared to standard of care

4.2 Secondary Objectives

- To determine the safety of the antiviral
- To determine the clinical benefit of the antiviral over placebo according to the WHO 7-point ordinal scale
- To determine the clinical benefit of the antiviral over placebo on time to resolution of clinical symptoms
- To determine the effect of the antiviral over placebo on biomarkers of inflammation and immune activation

5 OUTCOMES AND OUTCOME MEASURES

5.1 PRIMARY ENDPOINT

- Time to virological cure as defined by 2 successive throat (or combined nose/throat) swabs negative for SARS-CoV-2 by nucleic acid testing

5.2 SECONDARY ENDPOINTS

- Safety defined as all adverse events definitely, probably or possibly related to study treatment.
- Time from randomisation to an improvement of two points (from the status at randomisation) on the 7-point ordinal scale
    - The 7-point ordinal scale consists of the following categories: 1, not hospitalized with resumption of normal activities; 2, not hospitalized, but unable to resume normal activities; 3, hospitalised, not requiring supplemental oxygen; 4, hospitalised, requiring supplemental oxygen; 5, hospitalised, requiring nasal high-flow oxygen therapy, noninvasive mechanical ventilation, or both; 6, hospitalised, requiring ECMO, invasive mechanical ventilation, or both; and 7, death.
- Time from randomisation to resolution of clinical symptoms (fever, cough, shortness of breath, cough). Resolution defined as the start of the first 24 hour period when all symptoms are rated as mild or absent and remained this way for 24 hours
- Changes in SARS-CoV-2 viral load from nose/throat swabs from baseline over time
- Biomarkers taken as part of routine care including total lymphocyte count, CRP, Ferritin and LDH.
- Investigational markers of immune response such as markers of immune activation (e.g. IL-2R, IL-6, IL-10, GM-CSF, CXCL10, CCL2, TNF- alpha, HLA-DR+ expression on T-cells) and cellular and humoral (e.g. establishing T-and B-cell memory, development of neutralising antibodies) immune responses

6 EXPERIMENTAL DESIGN

6.1 STUDY SITE

We are aiming to recruit individuals at Alfred Health and Monash Health with additional sites added if there is the availability of a committed principal site investigator and site research team at other clinical services; and capacity to collect samples as per the protocol.

Sites will enrol individuals who are hospitalised and also individuals diagnosed through participating laboratories that are in the community.

6.2 STUDY DESIGN

This is a randomised placebo controlled phase II trial in COVID-19 infected individuals in hospital or community settings. Individuals will be randomised to the candidate antiviral or matched placebo and randomisation will be stratified according to whether the participant requires hospitalisation or not. This treatment will be given in addition to the usual standard of care in the participating hospital.

Adaptive design: The interim trial results will be monitored by an independent Safety Monitoring Committee (SMC) detailed in Section 10.8. The most important task for the SMC will be to assess whether the randomised comparison in the study have provided evidence of an antiviral effect (with a range of uncertainty around the results that is narrow enough) to impact treatment strategies. In such a circumstance, the SMC will inform the Trial Protocol Steering Committee who will amend the trial accordingly. New trial arms can be added as evidence emerges that other candidate therapeutics should be evaluated

6.3 DURATION OF STUDY

Eligible participants with confirmed COVID-19 infection will be randomised 1:1 to the first candidate antiviral Favipiravir or placebo in addition to standard of care. Participants will be given Favipiravir/Placebo for 14 days. Study participants can be either a hospital inpatient, at home in the community or a combination of inpatient / community. Participants will be followed for a minimum of 28 days. Participants have the option to provide additional blood samples to assess longer term immunological outcomes at additional visits up to 12 months post randomisation

6.4 STUDY IMPLEMENTATION TIMELINE

May 2020:	Study concept and design finalised
June 2020:	Study protocol finalised
July 2020:	Ethics submission to Alfred HREC
	Contracts and agreements (Alfred, Monash, participating sites)
	Enrolment commences, first participant first visit
Aug-October 2020	Enrolment
November 2020	Study report of first candidate antiviral

7 ELIGIBILITY CRITERIA

7.1 INCLUSION CRITERIA

- Provision of informed consent by the participant
- Age ≥18 years
- Confirmed SARS-CoV-2 by nucleic acid testing in the past 5 days
- COVID-19 related symptom (one or more of: fever, cough, sore throat, shortness of breath, fatigue, myalgia) initiation within 5 days

7.2 EXCLUSION CRITERIA

- Known allergy to the study medication

- Is on another clinical trial investigating an antiviral treatment for COVID-19
- Pregnancy or Breastfeeding
- Treating team deems enrolment in the study is not in the best interests of the patient
- Unable to provide consent
- Death is deemed to be imminent within the next 24 hours

.

7.3 TREATMENT DISCONTINUATION, PARTICIPANT WITHDRAWALS AND LOSSES TO FOLLOW-UP

7.3.1 TREATMENT DISCONTINUATION – PARTICIPANT REMAINS IN TRIAL

Participants who discontinue trial treatment will remain in the trial. The remaining trial procedures/visits should be completed as indicated by the trial protocol.

Participants may discontinue trial treatment for the following reasons:

- Participant request;
- Investigator decision to discontinue a participant from trial intervention if the participant:
    - is pregnant;
    - demonstrates significant non-compliance with the trial intervention;
    - experiences a serious or intolerable adverse event such that continued trial intervention would not be in the best interest of the participant;
    - requires medication that is prohibited by the protocol;
    - requires early discontinuation for any other reason.

The investigator may also withdraw all study participants from the trial if the trial is terminated early.

For the safety of all participants ceasing trial treatment, the protocol specified safety evaluations should be undertaken to capture new safety events and to assess existing, unresolved safety events. Information related to study withdrawal will be documented in the site assessment forms including the reason for withdrawal, date of withdrawal, and whether the participant or investigator made this decision. If withdrawal from the study is due to an adverse event, this will be followed up as detailed under adverse event reporting in this protocol.

The participant should remain in the trial for scheduled visits for trial assessments (follow-up) per protocol.

7.3.2 WITHDRAWAL OF CONSENT – PARTICIPANT WITHDRAWS FROM ALL TRIAL PARTICIPATION

Participants are free to withdraw from the trial at any time upon their request. Withdrawing from the trial will not affect their access to standard treatment or their relationship with the hospital and affiliated health care professionals.

For the safety of all participants ceasing trial treatment, reasonable efforts should be made to undertake protocol-specified <u>safety evaluations</u> to capture new safety events and to assess existing, unresolved safety events following withdrawal.

7.3.3 Losses to follow-up

A participant will be considered lost to follow-up if he or she fails to return for 2 scheduled visits and is unable to be contacted by the trial site staff. The following actions must be taken if a participant fails to return to the clinic for a required trial visit:

- The site will attempt to contact the participant and counsel the participant on the importance of maintaining the assigned visit schedule and ascertain if the participant wishes to and/or should continue in the trial;
- Before a participant is deemed lost to follow-up, the investigator or designee will make every effort to regain contact with the participant. These contact attempts should be documented in the participant's medical record or trial file;
- Should the participant continue to be unreachable, he or she will be considered to have withdrawn from the trial with a primary reason of lost to follow-up.

7.3.4 Definition of inclusion and replacement of participants

A participant is defined as included in the study when he or she has undergone randomisation. Any exclusion or withdrawal from the study prior to randomisation will be defined as screening failure.

Participants withdrawn from the study after randomisation before receiving the final doses of study drugs will be replaced at the discretion of the sponsor. After consultation between the principal investigator and at least one other member of the protocol steering committee, enrolment may be extended to replace participant(s) discontinued during the study.

7.3.5 Trial Closure

A participant is considered to have completed the trial if he or she has completed all phases of the trial including the last visit or the last scheduled procedure shown in the Schedule of Assessments.

The end of the trial is defined as completion of the last visit or procedure shown in the Schedule of Assessments in the trial at all sites. At this stage, the Sponsor will ensure that all HRECs and RGOs as well as all regulatory and funding bodies have been notified.

7.3.6 Temporary halt or early termination of the trial

This trial may be temporarily suspended or prematurely terminated if there is sufficient reasonable cause. If the trial is prematurely terminated or suspended, the Sponsor will promptly inform investigators, trial participants, HREC and RGO, funding and regulatory bodies, providing the reason(s) for the termination or suspension. Circumstances that may warrant termination or suspension include, but are not limited to:

- Determination of an unexpected, significant, or unacceptable risk to participants
- Insufficient compliance to protocol requirements;
- Demonstration of efficacy that would warrant stopping;

In the case of concerns about safety, protocol compliance or data quality, the trial may resume once the concerns have been addressed to the satisfaction of the sponsor, HREC, RGO, funding and/or regulatory bodies.

Specific criteria that would lead the protocol team to pause enrolment in the study and convene a safety monitoring committee (SMC) meetings are (See Section 13.8 for more detail on the SMC):

- Death on study occurs. A death on study will lead to immediate suspension of recruitment and a meeting of the SMC within 48 hours;
- One Grade 4 or two Grade 3 drug related adverse events or laboratory abnormalities reported in any arm.

7.4 PARTICIPANT CONSENT

Potential eligible participants are identified by the research team are flagged by the departmental notification system. For hospital inpatients the research team will approach the clinical staff to discuss the potential participants' ability to consent. Individuals with COVID-19 that are not hospital inpatients will be contacted by phone by a member of the research team. Consent can be obtained from either the clinical staff or from the research team and will ensure that the study participant is fully informed about the nature and objectives of the research and possible risks associated with participation. This includes answering any questions the participant may have throughout the study. The project aims will be outlined it will be explained that the individual's voluntary involvement will place no impact on their medical care. Unspecified consent is being sort for consent for the use of data or tissue in any future research.

Verbal consent will be obtained rather than written consent due to safety measures required to protect the research team as these participants are in isolation. The verbal informed consent must be clearly documented in the participants medical history – this must include the date and who obtained the consent, that a discussion occurred about the study, that the participant demonstrated an understanding of the study and the study requirements and that the participant is aware that the can withdraw at any time. A copy of the PICF must also be filed in the participants' medical history, this must be signed and personally dated, by the person who performed the informed consent. A copy of the signed informed consent form should be provided to the participant via e-mail or post.

For individuals that are recruited from the community, the site research team will confirm the potential participants' eligibility and ability to consent. The site research team will contact the participant by phone and explain the trial and identify if they are interested in participating. If they agree the site research staff member will e-mail the Community PICF or send them a link to view the Community PICF they will inform the potential participant that they will refer them to the Community Clinical Research Unit (CCRU) research team based at the Alfred Hospital who are managing the community participants. The CCRU researcher will contact the participant to confirm their interest and eligibility. The researcher must ensure that the study participant is fully informed about the nature and objectives of the research and possible risks associated with participation. This includes answering any questions the participant may have throughout the study. The project aims will be outlined the individual's voluntary involvement that will place no impact on their medical care. Unspecified consent is being sort for consent for the use of data or tissue in any future research.

Verbal consent will also be obtained from participants recruited from the community in the same way as outlined for hospitalised participants above.

8 STUDY PROCEDURES

8.1 SCHEDULE OF ACTIVITIES

10 visits including the baseline visit will be performed during the study with the option for additional follow-up visits if the need arises. A full schedule of activities is detailed in Table 1

Table 1: Protocol Activities

**PROTOCOL ACTIVITIES**															
	**Visit 1 Screening**	**Baseline**	**Visit 2**	**Visit 3**	**Visit 4**	**Visit 5**	**Visit 6**	**Visit 7**	**Visit 8**	**Visit 9**	**Visit 10**	**Visit 11 Optional**	**Visit 12 Optional**	**Visit 13 Optional**	**Unscheduled visit [c]**
**Visit window**	**24 Hour Window pre D1**	**D1**	**D2 +/- 1**	**D4 +/- 1**	**D6 +/- 1**	**D8 +/- 1**	**D10 +/- 1**	**D12 +/- 1**	**D14 +/- 1**	**D21 +/- 2**	**D28 +/- 2**	**D90 +/- 30**	**D180 +/- 30**	**D365 +/- 30**	
Informed consent	✓														
Medical history (incl/excl criteria)	✓														
Concomitant medications	✓		✓		✓		✓		✓	✓	✓	✓	✓		
Vital signs	✓[g]		✓[g]		✓[g]		✓[g]		✓[g]	✓[g]	✓[g]				
Clinical review including symptom, and Adverse event survey [b] (online if in community)	✓		✓		✓		✓		✓	✓	✓	✓	✓		✓
Antiviral candidate / placebo dispensed		✓													
Pregnancy test [a], – if applicable	✓														
FBE, UEC, LFT [d]	✓									✓[e]	✓[e]				
Results of blood tests taken as part of routine care if performed (e.g. FBE, UEC, LFT, CRP, D-dimer, LDH, ferritin) [f]	✓		✓		✓		✓		✓	✓	✓				
Nasopharyngeal swab for SARS-CoV-2 PCR (self-collection if in community)	✓		✓	✓	✓	✓	✓	✓	✓						✓
Plasma and PBMC isolation incl. storage (optional)	✓			✓[g]		✓[g]					✓	✓	✓	✓	✓

NOTES:

a = For women of childbearing potential. After Visit 1 (Day 0), urine pregnancy testing only should be performed if the participant reports non-compliance to birth control methods or if there is a question about potential pregnancy possibility

b =Includes symptom assessment, 7-point ordinal scale, frailty score and QOL

c = Optional for individuals unable to undertake visits in study windows

d = candidate antiviral specific

e = This bleed on one of these 2 visits

f = Only if inpatient and part of routine care
g =only for inpatients

8.2 RANDOMISATION

Randomisation will be done at the Alfred Hospital Clinical Trials Pharmacy using computer generated block-randomisation lists with 6 participants per block. Within each block half the participants will be randomised to the candidate antiviral and the other half to placebo. Randomisation is stratified by study site with participants enrolled in the community considered as a study site. Each study participant is provided with a participant randomisation number, which is recorded on the electronic case report form (eCRF). The randomisation code containing information on randomisation numbers and corresponding treatment allocation will be provided to the clinical trials pharmacy at the Alfred Hospital and maintained by an investigator independent of all study staff at Alfred Health. Study participants and study investigators will be blinded to treatment allocation.

8.3 VISIT 1 – SCREENING / BASELINE

Informed consent must be obtained before any study-specific procedures are performed. Research blood samples will occur on the same day consent is obtained. Screening safety bloods will be transported to the site enrolling the participant.

Any screening pathology tests that are listed ***and*** which was taken as part of standard of care (SOC) within 5 days of study baseline visit can be used to assess eligibility and does not need to be repeated specifically for this study, with the exception of pregnancy testing.

The following evaluations and procedures must be performed:

- Informed consent obtained;
- Review medical history to determine eligibility based on the inclusion/exclusion criteria including evidence of a diagnosis of COVID-19 based on a positive nucleic acid test for SARS-CoV-2 from a nasopharyngeal sample
- Demographic information („e.g; age, sex, ethnicity, postcode);
- COVID Risk factors (from date of +ve COVID19 test result: e.g.travel history last 30 days, contact with person known to be COVID infected)
- Review medications history (including concomitant medications prescription and over-the-counter)
- Clinical record review including signs and symptoms,
- Vital signs,
- Quality of life assessment
- Single combined Nose / Throat swab for SARS-CoV-2 nucleic acid testing
- If applicable collect blood for:
    - Full blood examination
    - Urea, electrolytes, creatinine,
    - Liver function tests
    - Pregnancy test
    - Plasma and PBMCs for storage - up to 4 x 9ml blood tubes (36 mL) (optional)

If the participant is a hospital inpatient this will be performed by study staff or ward staff as per standard hospital procedures

If the participant is not a hospital inpatient, then the CCRU research staff will attend the

residence of the participant to collect the blood samples listed above. Attendance at the home of an individual infected with COVID-19 will be in accordance with hospital procedures already in place to visit the home for care of these individuals. This includes the use of personal protective equipment (PPE) by the research co-ordinator and maintaining standards of infection prevention to prevent onward transmission of COVID-19.

Once a participant is successful in the screening process they will be randomised and the candidate antiviral/placebo will be dispensed.

Screen failures are defined as participants who consent to participate in the trial, but who are found, during the screening process, to be ineligible to continue in the trial.

8.3.1 VISITS 2, 4, 6, AND 8

The following information will be collected for entry into the eCRF:

- Concomitant medications
- Any adverse events or change in symptoms which have occurred since prior visit (for participants in the community they will have the option of completing this as an online survey);
- Vital signs if participant is still an inpatient
- Quality of life assessment
- Results of laboratory tests taken as part of routine care if performed (FBE, renal function, liver function, CRP, LDH, Ferritin, Lactate, Troponin, INR, APTT, d-dimer if these tests performed)

The following laboratory samples will be collected:

- Single combined Nose / Throat swab for SARS-CoV-2 nucleic acid testing

If the participant is a hospital inpatient this will be performed by study staff or ward staff as per standard hospital procedures

If the participant is a community participant the research staff will call or perform a telehealth visit to assess the participant's health status and will send a self-swab collection kit to the participant who will be given information on how to collect this swab per Australian government guidance https://www.health.gov.au/sites/default/files/documents/2020/06/phln-guidance-covid-19-swab-collection-upper-respiratory-specimen.pdf

8.3.2 VISITS 3, 5 AND 7

The following laboratory samples will be collected:

- Single combined Nose / Throat swab for SARS-CoV-2 nucleic acid testing
- Plasma and PBMCs for storage - up to 4 x 9ml blood tubes (36 mL) (optional visit 3 & 5)

If the participant is a hospital inpatient this will be performed by study staff or ward staff as per standard hospital procedures

If the participant is a community participant the research staff will call or perform a telehealth visit to assess the participant's health status and will send a self-swab collection kit to the participant who will be given information on how to collect this swab per Australian government guidance https://www.health.gov.au/sites/default/files/documents/2020/06/phln-guidance-covid-19-swab-collection-upper-respiratory-specimen.pdf

8.3.3 VISIT 9

The following information will be collected for entry into the eCRF:

- Concomitant medications
- Any adverse events or change in symptoms which have occurred since prior visit (for participants in the community they will have the option of completing this as an online survey);
- Vital signs if participant is still an inpatient
- Collect blood on this visit or Visit 10 for:
  - Full blood examination
  - Urea, electrolytes, creatinine,
  - Liver function tests
  - Plasma and PBMCs for storage - up to 4 x 9ml blood tubes (36 mL) (optional)
- Results of other laboratory tests taken as part of routine care if performed (CRP, LDH, Ferritin, Lactate, Troponin, INR, APTT, d-dimer if these tests performed)

If the participant is not a hospital inpatient and has been cleared from self isolation by the local health department the participant has the option of attending the study site or a local pathology service to collect bloods

8.3.4 VISIT 10

The following information will be collected for entry into the Case report form (CRF):

- Concomitant medications
- Any adverse events or change in symptoms which have occurred since prior visit (for participants in the community they will have the option of completing this as an online survey);
- Vital signs if participant is still an inpatient
- Clinical review including signs and symptoms,
- Quality of life assessment
- Collect blood on this visit if not already done so on Visit 9:
  - Full blood examination
  - Urea, electrolytes, creatinine,
  - Liver function tests
  - Plasma and PBMCs for storage - up to 4 x 9ml blood tubes (36 mL) (optional)
- Results of other laboratory tests taken as part of routine care if performed (CRP, LDH, Ferritin, Lactate, Troponin, INR, APTT, d-dimer if these tests performed)

If the participant is not a hospital inpatient and has been cleared from self isolation by the local health department the participant has the option of attending the study site or a local pathology service to collect bloods

8.3.5 VISITS 11, 12 AND 13 (OPTIONAL)

The following evaluations and procedures must be performed:

- Concomitant medications

- Any adverse events or change in symptoms which have occurred since prior visit (for participants in the community they will have the option of completing this as an online survey);
- Vital signs if participant is still an inpatient
- Clinical review including signs and symptoms,
- Quality of life assessment
- Collect blood for:
  - Plasma and PBMCs for storage - up to 4 x 9ml blood tubes (36 mL) (optional)

The participant has the option of attending the study site or a local pathology service to collect bloods

9 LABORATORY SAMPLES

9.1 BLOOD SAMPLES

Al blood samples will be from standard of care apart from additional screening blood samples if individuals are recruited in the community (Full blood examination, renal and hepatic function) at screening and 3-4 weeks after commencing study drug. The only other additional blood samples are the optional bloods or storage of plasma and PBMCs (36 ml of peripheral blood) which also occurs at screening, 3-4 weeks after commencing study drug and then at day 3-5, 7-9, 28, 90, 180 and 365 days after commencing study drug

Each sample will be labelled with the patient's unique study code; visit number and date of collection. Ideally these samples are collected at the time of routine blood draws.

Optional storage blood samples will need to be collected before 3 pm and to be transported to the Alfred Health Department of Infectious Diseases laboratory, Commercial Rd, Melbourne, within 4 hours from time of collection. The exception is samples from Monash Health which will be stored at the Monash Biobank, MHTP, Clayton. These samples will be stored for future analysis related to COVID-19 research, in the COVID 19 Biobank including genetic testing and Human Leukocyte Antigen (HLA) testing, conditional on adherence to local regulations and ethical guidelines. Genetic testing is for research purpose and not suitable for clinical purposes and therefore the results, no matter what the findings, will not be returned to the participant.

For the type of collection tubes to be used please refer to the VIRCO Trial Laboratory Manual.

9.2 COMBINED NOSE / THROAT SWAB

A single combined nose and throat swabs will be collected on visits 1 – 8. The swab is collected and transported in a viral medium, which will be labelled with the patient's unique study code, visit number and date of collection. The samples need to be collected before 3 pm and transported within 4 hours of time of collection to the laboratory. Participants will be provided with packaging and courier of samples will be performed at no cost to the individual.

9.3 SAMPLE PROCESSING AND STORAGE

The collection, processing, storage and transport of biological samples will follow local standard operating procedures and regulations for handling and transporting clinical specimens containing infectious materials. Shipping material will permit the samples to be transported safely, paperwork that accompanies the samples identify the samples as part of COVID19 Biobank.

The laboratories receiving the Biobank samples are equipped to receive biological specimens or materials (biologicals), they have the appropriate practices and equipment to manage the risks associated with those biologicals. Staff are procedurally trained to process all biologicals as potentially hazardous or infectious.

10 SAFETY ASSESSMENTS

10.1 ADVERSE EVENT (AE)

An AE is any untoward medical occurrence in a patient or clinical investigation participant administered a pharmaceutical product that does not necessarily have a causal relationship with this treatment. An AE can therefore be any unfavourable and unintended sign (including an abnormal laboratory finding – see below), symptom, or disease temporally associated with the use of the investigational drug, whether or not causally linked to the investigational drug.

An abnormal laboratory value or test result constitutes an adverse event only if

- It is associated with clinical signs or symptoms;
- It leads to a change in study drug dosing or discontinuation from the study;
- It requires additional diagnostic testing or medical/surgical intervention;
- It is considered to be an adverse event by the investigator.

In addition, all cases of drug-drug interaction, pregnancy (with or without outcome), paternal exposure, lactation, overdose, drug abuse and misuse, drug maladministration or accidental exposure and dispensing errors are collected, and data based even if no adverse event has been reported.

All adverse events will be reported in accordance with the principles of Good Clinical Practice and the latest requirements of the Medicines for Human Use (Clinical Trials) Regulations.

10.2 SERIOUS ADVERSE EVENT (SAE)

A SAE is defined as any untoward medical occurrence that at any dose:

- Results in death;
- Is life-threatening;
- Results in persistent or significant disability/incapacity;
- Constitutes a congenital anomaly/birth defect;

- Requires inpatient hospitalization or prolongation of existing hospitalization, *unless hospitalization is for:*
    - Routine treatment or monitoring of the studied indication, not associated with any deterioration in condition;
    - Elective or pre-planned treatment for a pre-existing condition that is unrelated to the indication under study and has not worsened since the start of the study drug;
    - Treatment on an emergency outpatient basis for an event not fulfilling any of the definitions of a SAE given above and not resulting in hospital admission;
    - Social reasons and respite care in the absence of any deterioration in the patient's general condition.
- Is medically significant, i.e. defined as an event that jeopardizes the patient or may require medical or surgical intervention to prevent one of the outcomes listed above

Unlike routine safety assessments, SAEs are monitored continuously and have special reporting. All SAEs that occur from randomisation up to until 28 days following the last dose of an investigational product will be documented and reported; SAEs that occur thereafter will only be documented and reported if they are deemed to be at least possibly related to any investigational product.

10.3 Suspected Unexpected Serious Adverse Reaction (SUSAR)

Consider a SUSAR as any SAE that is both suspected to be related to the trial treatment and is unexpected (i.e. not consistent with the available safety information in the Investigator's Brochure (for unapproved products) /approved Product Information.

10.4 Assessment of adverse events

Any study participant who receives at least one dose of investigational drug/placebo will be included in the evaluation for safety. Safety assessment is done by recording of all patient-reported AEs and SAEs. For each AE/SAE the relationship to the investigational drug will be evaluated and the severity graded according to the Division of AIDS (DAIDS) Table for Grading the Severity of Adult and Pediatric Adverse Events, Corrected Version 2.1 (July 2017) (http://rsc.tech-res.com/clinical-research-sites/safety-reporting/daids-grading-tables).

Any severe adverse events (requiring prolongation of hospital care or hospitalisation) considered by the investigators of the institution due directly to a study procedures will be reported to their own institution as part of governance process. The Alfred Health investigators: Dr James McMahon or Dr Jillian Lau should be informed of all SAE's reported to site governance within 24 hours, for notification to Alfred Hospital Ethics Committee.

10.5 Recording of Adverse Events

At each contact with the participant, information is sought on adverse events by specific questioning and, as appropriate, by examination. Information on all adverse events should be recorded immediately in the source document, and also in the appropriate adverse event module of the eCRF. All clearly related signs, symptoms, and abnormal diagnostic

procedures results should be recorded in the source document, though they should be grouped under one diagnosis.

All adverse events (non-serious and serious) will be captured from the time of administration of the investigational products until the last study visit and the clinical course of each event should be followed until resolution, stabilization, or until it has been determined that the study treatment or participation is not the cause. Serious adverse events that are still on-going at the end of the study period must be followed up to determine the final outcome. Any serious adverse event that occurs after the study period and is considered to be possibly related to the study treatment or study participation should be recorded and reported immediately.

For the purposes of this trial, the investigator is responsible for recording all adverse events, regardless of their relationship to the trial drug, with the following exceptions:

- Conditions that are present at screening and do not deteriorate will not be considered adverse events.
- Abnormal laboratory values will not be considered adverse events unless deemed clinically significant by the investigator and documented as such.

The AE will be described in the source documents and captured on the CRF and will include:

- A description of the AE
- The onset date, duration, date of resolution
- Severity (mild, moderate or severe – what is the impact on the participant's daily life?)
- Seriousness (i.e. is it an SAE?)
- Any action taken, (e.g. treatment, follow-up tests)
- The outcome (recovery, death, continuing, worsening)
- The likelihood of the relationship of the AE to the trial treatment (Unrelated, Possible, Probable, Definite)

Changes in the severity of an AE will be reported. AEs characterised as intermittent will be documented for each episode.

All AEs will be followed to adequate resolution, where possible. All AEs must be graded according to the Division of AIDS (DAIDS) Table for Grading the Severity of Adult and Pediatric Adverse Events, Corrected Version 2.1 (July 2017) (http://rsc.tech-res.com/clinical-research-sites/safety-reporting/daids-grading-tables). If the adverse event is not specifically identified in the DAIDS grading table, or only signs and symptoms are known (not a diagnosis), then all adverse events will be graded in the following manner:

- Grade 1 (Mild): Events require minimal or no treatment and do not interfere with the participant's daily activities;
- Grade 2 (Moderate): Events result in a low level of inconvenience or concern. Moderate events may cause some interference with functioning;
- Grade 3 (Severe): Events interrupt a participant's usual daily activity and may require systemic drug therapy or other treatment. Severe events are usually incapacitating;
- Grade 4 (Life-threatening): Any adverse drug experience that places the participant, in the view of the investigator, at immediate risk of death from the reaction as it occurred

(i.e., it does not include a reaction that had it occurred in a more severe form, might have caused death);

- Grade 5 (Death).

10.6 Assessing the relatedness (causality) of a participant's AE

All adverse events must have their relationship to trial intervention assessed by the investigator who evaluates the adverse event based on temporal relationship and his/her clinical judgment. The degree of certainty about causality will be graded using the categories below. In a clinical trial, the trial product should always be suspected.

The relationship of the event to the trial intervention will be assessed as follows:

- **Unrelated:** There is no association between the trial intervention and the reported event. AEs in this category do not have a reasonable temporal relationship to exposure to the test product or can be explained by a commonly occurring alternative aetiology.
- **Possible:** The event could have cause or contributed to the AE. AEs in this category follow a reasonable temporal sequence from the time of exposure to the intervention and/or follow a known response pattern to the test article but could also have been produced by other factors.
- **Probable:** The association of the event with the trial intervention seems likely. AEs in this category follow a reasonable temporal sequence from the time of exposure to the test product and are consistent with the known pharmacological action of the drug, known or previously reported adverse reactions to the drug or class of drugs, or judgement based on the investigators clinical experience.
- **Definite:** The AE is a consequence of administration of the trial intervention. AEs in the category cannot be explained by concurrent illness, progression of disease state or concurrent medication reaction. Such events may be widely documented as having an association with the test product or that they occur after rechallenge.

10.7 Reporting of Serious Adverse Events

The Investigator is responsible for expedited reporting (within 24 hours of becoming aware of the event) to the Sponsor the following local safety events:

- SUSARs
- All SAEs

The Site Principal Investigator is responsible for reporting SAEs (including SUSARs) to the Sponsor and HREC as soon as possible but within 24 hours of the first knowledge of the event. These Reports to the sponsor should be submitted using the SAE form in REDCap and notifying HREC by standard reporting methods at the clinical site.

Information about all SAEs is collected and recorded on a Serious Adverse Event Report Form. The minimum amount of information that the site investigator must complete on the initial report is:

- title of the event – avoid colloquialisms or abbreviations;
- date started;
- reason the event is considered an SAE;

- causality relationship to investigational drug/device.

The eCRF report is then to be printed before being signed and dated by the investigator. The signed form is then to be uploaded into REDCap. The principal investigator or designee should email sponsor representatives (below) to the inform them that a SAE report has been uploaded:

Coordinating PI – James McMahon: james.mcmahon@monash.edu
Study manager – Janine Roney: j.roney@alfred.org.au

All collaborators who are responsible for safety reporting on products used in this trial will be informed within 72 hours of any SAE becoming known to the Site Principal Investigator and sent a copy of an SAE report form. The Sponsor is responsible for reporting to all study stakeholders.

The original copy of the SAE Report Form must be maintained at the study site.

10.8 Safety Monitoring Committee

A Safety Monitoring Committee will be formed from clinician researchers with expertise in the conduct of randomised clinical trials to assess the progress of the clinical trial including safety data and during the trial the SMC should review:

- Study progress and safety data;
- Adherence to the protocol;
- Factors that might affect the study outcome or feasibility or compromise the trial data (e.g. slow accrual, poor data quality/timeliness, protocol violations, loss to follow-up);
- Data relevant to continuing or amending the study. The SMC will assess whether the randomised comparison has provided evidence of an antiviral effect (with a range of uncertainty around the results that is narrow enough).

The committee will then make a recommendation to the Trial Protocol Steering Committee whether to continue, modify or stop the study.

10.8.1 Interim review by Safety Monitoring Committee

An interim review of safety data by the SMC and Principal Investigator will occur after the first 20 participants have completed dosing of drug. A report summarising clinical and laboratory data will be provided to the SMC. The decision to proceed for the remainder of the participants will then be taken after a meeting of the SMC.

Data presented to the SMC will include summaries of baseline patient characteristics and details of participant enrolment

Safety data to be presented to the SMC will include:

- All Serious Adverse Events (SAEs), their expectedness and relationship to study drug. and suspected unexpected serious adverse reactions (SUSARs);
- All grade 3 or 4 adverse events;
- All immune related adverse events;
- All adverse events leading to premature cessation of the study drug;

- Summaries of changes in safety bloods;
- Details of any deaths.

The SMC may ask for further details or summaries of safety data as they regard appropriate

The protocol team will pause enrolment/dosing in the study and convene additional SMC meetings if:

- Death on study occurs. A death on study will lead to immediate suspension of recruitment and a meeting of the SMC within 48 hours;
- One Grade 4 or two Grade 3 drug related adverse events or laboratory abnormalities reported.
- At any time as requested by the investigators.

After the meeting for assessing the first 20 participants meetings will occur at least every 6 months and more frequently if this is deemed necessary by the SMC and/or PSC

10.8.2 SMC RECOMMENDATIONS

At each SMC review of safety and efficacy data, the SMC will recommend to the Protocol Steering Committee one of the following courses of action:

- Continue the study without modification;
- Pause enrolment pending either resolution of specific issues or amendment of the protocol as specified;
- Terminate the study.

Verbal communication of the SMC recommendation will be made to the principal investigator within 24 hours of the SMC meeting, with formal written communication to follow within one week.

10.9 SPONSOR REPORTING PROCEDURES

The Sponsor must act in accordance with the NHMRC's 'Safety monitoring and reporting in clinical trials involving therapeutic goods' (November 2016) and any additional requirements of the approving HREC. All safety reports must clarify the impact of the safety event on participant safety, trial conduct and trial documentation.

The Sponsor will report SUSARs to the TGA as follows:

1. Fatal or life-threatening SUSARs immediately, but no later than 7 calendar days after being made aware of the issue (follow up info within a further 8 calendar days)
2. All other SUSARs no later than 15 calendar days of being made aware of the issue

The Sponsor is responsible for providing the additional safety information to the approving HREC:

1. Provide an annual safety report, including a summary of the evolving safety profile of the trial
2. Provide any updated Product Information/Investigator's Brochure for the investigational products (if applicable)

The Sponsor is also responsible for providing any updated Product Information/Investigator's Brochure to Investigators.

10.10 Risk management and safety

The potential harms, risks and/or inconveniences associated with participation in this study will be that of exposure to the candidate antiviral (In the first instance Favipiravir), the risk of phlebotomy, the risk of additional nose/throat swabs, the risk of pregnancy in women of childbearing potential and the inconveniences associated with study visits. Specific risks related to these study procedures are listed below. To monitor the safety and ensure the well-being of study participants, monitoring and handling of adverse events will be done at study visits as detailed in section 8 of this protocol. In the event of adverse effects, participants will have medical care available through the hospital site of recruitment.

In the event of clinical deterioration or adverse events that require hospitalisation for people enrolled in the community they will be referred back to the hospital site that originally diagnosed the participant. This will occur as the hospital site originally diagnosing the participant will be able to collect further safety data and determine the outcome for that participant. If presentation or hospitalisation at the original site diagnosing the participant is not possible the Community Clinical Research Unit will facilitate presentation at a hospital that is able to care for the participant. Any medical testing and evaluation undertaken by study investigators to follow up abnormal findings will also be at no cost to participants

To reduce risk of infection transmission, research staff will use personal protective equipment (PPE) when obtaining verbal consent and collecting any samples of participants whilst they are in isolation. Noting that all efforts will be made to have study samples collected at the same time as clinical care sample collection if that is also occurring.

10.10.1 Candidate Antiviral

Each candidate Antiviral will have its own set of potential risks. Please refer to Appendices for discussion of safety for candidate antivirals.

10.10.2 Pregnancy

Any female participant who becomes pregnant during the study must be immediately withdrawn from study drugs to eliminate further exposure to the embryo/foetus.

The Investigator will also report this event to the HREC within 24 hours of becoming aware of the pregnancy. The investigator must request the participant's permission to query pregnancy outcome and follow each participant to determine the outcome of the pregnancy. When permission is received, participants will continue to be followed for safety assessments to trial discharge per protocol. The Investigator will monitor the participant and follow the outcome of the pregnancy. If the end of the pregnancy occurs after the trial has been completed, the outcome will be reported directly to the sponsor.

Pregnancy complications and elective terminations for medical reasons must be reported as an AE or SAE. Spontaneous abortions must be reported as an SAE. Any SAE occurring in association with a pregnancy brought to the investigator's attention after the participant has completed the study and considered by the investigator as possibly related to the investigational product, must be promptly reported to Sponsor.

Male participants will be instructed through the Informed Consent Form to immediately inform the Investigator if their partner becomes pregnant until the end of follow-up period. Attempts will be made to collect and report details of the course and outcome of any pregnancy in the partner of a male participant exposed to study drug with the consent of the pregnant partner.

10.10.3 Phlebotomy

The risks associated with phlebotomy include local pain, bruising, occasional light-headedness and fainting. Sometimes the blood vessel may swell, or blood may clot in the blood vessel, or the tissue nearby could become inflamed. Rarely, there could be a minor infection or bleeding. If this happens it can be easily treated. To minimise the risk blood will be taken by experienced staff.

10.10.4 Combined Nose / Throat Swabs

There are additional combined nose / throat swabs above standard of care that may cause some discomfort. To minimise any discomfort for hospitalised patients this will be done by experienced staff.

11 Statistical analysis

11.1 Sample size calculation

The sample size calculation was based on estimates from two published studies. In one randomised clinical trial of 236 patients with COVID-19, 61% of people receiving Favipiravir met the primary clinical recovery endpoint by day 7, compared to 52% of people receiving Arbidol. (Chen medRxiv doi.org/10.1101/2020.03.17.20037432). In a non-randomised study of 80 people, 90% of individuals receiving Favipiravir reached viral clearance from respiratory tract samples compared to 57% of individuals receiving lopinavir/ritonavir, with a significantly faster rate of clearance in patients who received Favipiravir (Hazard Ratio (HR) 3.43, 95% CI 1.16 to 10.1) (Cai et al, 2020). Allowing for more conservative estimates, we assumed 80% of patients on Favipiravir and 60% of patients in the placebo arm would reach virological cure by study end, with twice as fast a rate of cure occurring in the Favipiravir arm (HR 2.0). Assuming an alpha of 0.05, 86 participants in each arm would allow a log rank comparison with 80% power. Allowing for 10% LFU, this study aims to recruit 190 people (95/arm).

11.2 Analysis plan

11.2.1 Analysis Populations

The following analysis populations will be defined for the study:

- Intent-to-treat (ITT) population – All participants randomised to study drug
- Safety population – All ITT participants who received one dose or more of study drug
- Modified intent-to-treat (MITT) – All participants of the safety population that have an assessment of SARS-CoV-2 by nasopharyngeal PCR after randomisation and are confirmed COVID-19 positive
- Per protocol (PP) population – All MITT participants who adhere to relevant study procedures and have an outcome assessment.

11.2.2 Baseline Data

Continuous variables at baseline will be summarized using mean and confidence intervals (CI) or median and inter quartile ranges (IQR), as appropriate. Baseline numbers and percentage distribution will be quantified for categorical variables.

11.2.3 Primary endpoint

The primary outcome measure is the time to virological cure as defined by the first of 2 successive throat (or combined nose/throat) swabs negative for SARS-CoV-2 by nucleic acid testing during the 14 days after enrolment

The primary outcome will be compared between the treatment and placebo arm using a Kaplan-Meier time to event analysis with virological cure as the endpoint in an ITT population.

Exploratory analyses include:

- Log-rank test of the time to virological cure with the antiviral as compared with placebo in the MITT and PP populations
- A stratified log-rank test of the time to virological cure with the antiviral as compared with placebo with stratification by need for inpatient hospitalisation
- Uni- and multivariable cox proportional hazards model to identify factors predicting a negative nasopharyngeal swab by nucleic acid testing during the 14 days after enrolment.

11.2.4 Secondary endpoints

Safety will be assessed by comparing AEs, treatment related AEs, SAEs and treatment related SAEs between the those receiving the antiviral or placebo in the safety population. The difference in proportions of AEs and treatment related AEs will be compared by chi square tests for the different AEs and by AEs grouped by organ system. An overall summary of all AEs will be presented by treatment group, with participant counts and percentages of participants with the event.

Time to event endpoints will be compared between the antiviral and placebo groups using similar methods as for the primary endpoint; these will include time to: an improvement of two points (from the status at randomisation) on the 7-point ordinal scale, time to resolution of clinical symptoms and time to improvement in biomarkers of inflammation and immune activation.

The fractional change in SARS-CoV-2 viral load from nose/throat swabs will be assessed at each timepoint that a swab is taken from Baseline to Day 14 and the rate of change will be compared between treatment groups.

12 Ethics and Dissemination

12.1 Research Ethics Approval & Local Governance Authorisation

This protocol and the informed consent document and any subsequent amendments will be reviewed and approved by the human research ethics committee (HREC) prior to commencing the research. A letter of protocol approval by HREC will be obtained prior to the commencement of the study, as well as approval for other trial documents requiring HREC review.

For any other participating institutions, they will also obtain institutional governance authorisation for the research and associated HREC-approved documents. A letter of authorisation will be obtained from the research governance officer prior to the commencement of the research at that institution. Institutional governance authorisation for any subsequent HREC-approved amendments will be obtained prior to implementation at each site.

12.2 CONFIDENTIALITY

Participant confidentiality is strictly held in trust by the participating investigators, research staff, and the sponsoring institution and their agents. This confidentiality is extended to cover testing of biological samples and genetic tests in addition to the clinical information relating to participants.
Study samples before being transferred into the Biobank are confirmed to have no patient identifying information and that they are labelled with their unique Biobank number that will be linked to the participants study number.

The trial protocol, documentation, data and all other information generated will be held in strict confidence. No information concerning the study, or the data will be released to any unauthorised third party, without prior written approval of the sponsoring institution.
If any laboratory specimens, evaluation forms, reports or other records leave the site they will be identified only by the Participant Identification Number to maintain participant confidentiality.

Clinical information will not be released without written permission of the participant, except as necessary for monitoring by HREC or regulatory agencies.

13 DATA COLLECTION, PROCESSING AND STORAGE

13.1 SOURCE DATA

Source data are all information, original records of clinical findings, observations, or other activities in a clinical project necessary for the reconstruction and evaluation of the study. Electronic source data are data initially recorded in electronic form.

Examples of electronic or hardcopy documents that contain source data are: medical records [whether paper or via an Electronic Medical Record (EMR)]; photographs; pharmacy dispensing and other records; laboratory results; ECGs and reports; and imaging scans and reports.

13.2 DATA CAPTURE METHODS AND STORAGE

REDCap is hosted on the Alfred Health/ Monash University infrastructure and is participant to the same security and backup regimen as other systems (e.g. the network file servers). Data is backed up nightly to a local backup server, with a monthly backup taken to tape and stored offsite. All data transmissions between users and the REDCap server are encrypted. Regular data quality checks, such as automatic range checks, will be performed to identify data that appear inconsistent, incomplete, or inaccurate.

Access to REDCap is via an Alfred Health user account or for external collaborators via a REDCap user account created by the system administrator. The permissions granted to each user within each REDCap project is controlled by and is the responsibility of the project manager. REDCap has functionality that makes adding and removing users and managing user permissions straightforward. REDCap maintains an audit trail of data create/update/delete events that is accessible to project users that are granted permission to view it.

13.3 RECORD RETENTION

Data must be kept indefinitely. Electronic data will be stored on a password protected network drive located at Alfred Health. Identifiable data eg, names, contact details will be stored separately to other study data. Study data will be stored in a re-identifiable coded format. Data collected through record linkage will have identifiers replaced by the coded study number immediately upon receipt. Only researchers approved for this project will have access to the study data with levels of access according to role in the project. Paper consent forms will be stored in locked secure access restricted office.

Records should not be destroyed without the written consent of the Sponsor. It is the responsibility of the Sponsor to inform site investigators when these documents no longer need to be retained.

13.4 STORAGE OF BIOLOGICAL MATERIAL AND GENETIC TESTING

Blood samples can be collected for PBMC and plasma storage at some study visits as an optional procedure as described in section 8 of this protocol. These additional blood samples and then Nasal/Throat swabs collected for the trial will be stored as indicated in section 9 of the protocol.

All samples will be stored in accordance with NATA and ISO guidelines. The storage freezer where samples will be stored at -80 degrees C, is locked and monitored for temperature excursions, which lead to alarm activation. Stored samples may be used for future analyses related to COVID-19 research, including genetic testing and HLA typing, conditioned on adherence to local regulations and ethical guidelines. Genetic testing is for research purpose and not suitable for clinical purposes and therefore the results, no matter what the findings, will not be returned to the participant.

Samples will be stored at the Department of Infectious Diseases, Alfred Health for all sites except samples collected from patients at Monash Health which will be stored at the Monash Biobank, MHTP, Clayton. All samples will only be accessible for research following approval by the Protocol Steering Committee and HREC approval.
There is a possibility that sample/data may be sent trans-border in the future, as research that occurs for COVID-19 may entail collaboration with investigators outside Victoria or

Australia. This will only occur with approval from the PSC, who will decide the scientific merit of sending samples trans-border and HREC approval.

All applications for use of these samples require approval by at least 3 steering committee members. If 3 members are not available, other listed investigators on the protocol may give approval. Applications must demonstrate that they have ethical approval to conduct research on these samples. Any researchers outside Alfred Health must provide the appropriate agreement such as a Materials Transfer Agreement for access.

The address where these samples are stored is:
Alfred Health
85 Commercial Road
Melbourne VIC 3004
Australia

Contact: Dr. James H. McMahon; Head Clinical Research Unit,
Department of Infectious Diseases
Alfred Hospital, Melbourne, Australia
Email: james.mcmahon@monash.edu

For Monash Health Samples
Monash Biobank, MHTP, Clayton
Monash Health Translational Precinct
246 Clayton Rd, Clayton Victoria, 3168

Contact: A/Prof. Benjamin A Rogers, MBBS PhD FRACP
Infectious Disease Physician,
Monash Health, Clayton. Australia
Email: ben.rogers@monash.edu

14 Publication of Study

Research findings from this study will be published in a timely manner in international peer-reviewed journals. The coordinating principal investigator will have full control over the content of such publications.

15 References

1. Furuta Y, Komeno T, Nakamura T. Favipiravir (T-705), a broad spectrum inhibitor of viral RNA polymerase. Proc Jpn Acad Ser B Phys Biol Sci 2017;93:449-63.
2. Interactive Clinical Trial Tool. 2020. at https://iddo.cognitive.city/public/overview.)
3. Furuta Y, Takahashi K, Kuno-Maekawa M, et al. Mechanism of action of T-705 against influenza virus. Antimicrob Agents Chemother 2005;49:981-6.
4. Bai CQ, Mu JS, Kargbo D, et al. Clinical and Virological Characteristics of Ebola Virus Disease Patients Treated With Favipiravir (T-705)-Sierra Leone, 2014. Clin Infect Dis 2016;63:1288-94.

5. Oestereich L, Ludtke A, Wurr S, Rieger T, Munoz-Fontela C, Gunther S. Successful treatment of advanced Ebola virus infection with T-705 (favipiravir) in a small animal model. Antiviral Res 2014;105:17-21.
6. Wang M, Cao R, Zhang L, et al. Remdesivir and chloroquine effectively inhibit the recently emerged novel coronavirus (2019-nCoV) in vitro. Cell Res 2020;30:269-71.
7. FUJIFILM Toyama Chemical Co. Avigan Package Insert 7th Version English2019.
8. Cai Q, Yang M, Liu D, et al. Experimental Treatment with Favipiravir for COVID-19: An Open-Label Control Study. Engineering (Beijing) 2020.
9. Favipiravir versus Arbidol for COVID-19: A Randomized Clinical Trial. 2020. at https://www.medrxiv.org/content/10.1101/2020.03.17.20037432v4.)
10. COVID-19 Drug Interactions. 2020. at https://www.covid19-druginteractions.org.)
11. FUJIFILM Pharmaceuticals. Favipiravir Investigator's Brochure–Twelfth Edition (US Version).
12. Sissoko D, Laouenan C, Folkesson E, et al. Experimental Treatment with Favipiravir for Ebola Virus Disease (the JIKI Trial): A Historically Controlled, Single-Arm Proof-of-Concept Trial in Guinea. PLoS Med 2016;13:e1001967.

16 APPENDICES

16.1 APPENDIX A – CANDIDATE ANTIVIRALS

16.2 FAVIPIRAVIR

16.2.1 DOSING

1800 mg Favipiravir twice daily on Day 1 followed by 800 mg Favipiravir twice daily for the next 13 days.

16.2.2 OVERVIEW OF FAVIPIRAVIR

Favipiravir, is a small molecule anti-viral with activity against RNA viruses, including rabies, Ebola, Lassa, and coronaviruses. It has been studied in clinical trials for influenza, Severe Fever with Thrombocytopenia virus, Ebola, and has been used under compassionate release for Ebola, rabies, Lassa fever, norovirus and COVID-19.[1] It has been studied in over 40 clinical trials globally for the treatment of multiple RNA viruses, principally influenza and Ebola in more than 3100 subjects. It is currently being studied in 11 randomised clinical trials of COVID-19 infection globally.[2]

Host cellular enzymes convert Favipiravir to T-705 ribosyl triphosphate (T-705RTP), which selectively inhibits viral RNA polymerase. T-705-RTP competes with purine nucleosides and interferes with viral replication by incorporation into the virus RNA and thus, potentially inhibiting the RNA dependent RNA polymerase (RdRp) of RNA viruses.[3] Favipiravir has inhibitory activity against a broad spectrum of influenza A, B, and C viruses, including strains with poor susceptibility to amantadine hydrochloride or oseltamivir. Favipiravir has been approved in Japan for the treatment of uncomplicated influenza, when currently available anti-influenza medications are deemed to be ineffective. Favipiravir has been studied in Ebola virus diseases where it demonstrated higher EC50s (67 µM) in Vero E6 cell models but was 10% effective in an Ebola mouse model preventing lethal outcome in all treated animals and rapid viral clearance. It also suggested clinical benefit in a small uncontrolled clinical trial with 35 recipients receiving Favipiravir having 35% mortality, compared to 56% mortality in 85 historical controls.[4,5]

Favipiravir has activity against SARS-CoV-2 in vitro with a half maximal effective concentration or EC_{50} of 61.88 µM (9.7 µg/ml).[6] While this is higher than other nucleoside analogues such as Remdesivir,[6] Favipiravir has demonstrated mean daily trough levels over 20 µg/ml using the same dosing as proposed in this trial to treat COVID-19.[7]

Two clinical trials have been reported on the use of Favipiravir. The first a non-randomised trial of 80 patients in Shenzhen, China reported that the 35 patients in the favipiravir arm demonstrated significantly shorter viral clearance time as compared to 45 patients in the control arm (median 4 days vs. 11 days) with a higher rate of improvement in chest X-ray images in the Favipiravir arm (91 vs. 62%).[8] In another non peer-reviewed randomised clinical trial of 236 people, 61% of people receiving Favipiravir met the primary clinical recovery endpoint by day 7, compared to 52% of people receiving Arbidol.[9]

These in vitro and early clinical reports combined with oral dosing provide a strong rationale to trial this agent to treat COVID-19 infection. Importantly Favipiravir has an excellent safety profile in over 6 years of clinical use. There is a transient elevation of uric acid that resolve with cessation of dosing in < 5% of individuals. Other adverse events reported are diarrhoea in < 5% of individuals and elevations of liver enzymes and reductions in white cell count in < 1% of individuals.[7]

16.2.3 Study Design

Individuals will be randomised to Favipiravir or matched placebo and randomisation will be stratified according to whether the participant requires hospitalisation or not. This treatment will be given in addition to the usual standard of care in the participating hospital (Figure 1)

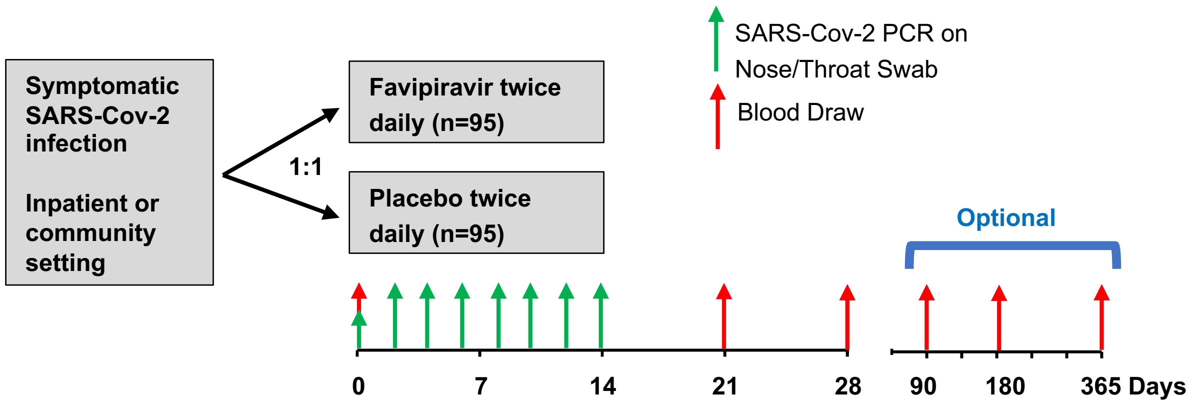

Figure 1. Study Design

16.2.4 Duration of Study

Eligible participants with confirmed COVID-19 infection will be randomised 1:1 to Favipiravir or placebo in addition to standard of care. Participants are given Favipiravir /Placebo for 14 days. Study participants can be either a hospital inpatient, at home in the community or a combination of inpatient / community.. Participants will be followed for a minimum of 28 days. Participants have the option to provide additional blood samples to assess longer term immunological outcomes at additional visits up to 12 months post randomisation

16.2.5 Additional Inclusion Criteria

- Female patients of childbearing potential must have a negative pregnancy test at Screening. Female patients of childbearing potential and fertile male patients who are sexually active with a female of childbearing potential must use highly effective methods of contraception throughout the study and for 1 week following the last dose of study treatment.

16.2.6 Additional Exclusion Criteria

- Pregnancy or Breastfeeding
- Patients with severe hepatic dysfunction equivalent to Grade C in the Child-Pugh classification
- Patients with renal impairment requiring dialysis

16.2.7 ADVERSE EFFECTS

Favipiravir has been studied in clinical trials in over 3100 study participants and is generally safe and well tolerated. More specifically Favipiravir has been well tolerated in studies in adults and elderly participants with uncomplicated influenza. A consistent safety profile composed of relatively low frequencies of mild to moderate adverse events (AEs) clustering around the system organ classes of gastrointestinal disorders, investigations, and infections and infestations has been characterized. Mild to moderate transient, asymptomatic elevations in serum uric acid and mild to moderate diarrhea are the two most common AEs known to occur with favipiravir occurring in < 5% of individuals (Table 2).[7]

In double blind studies, the adverse event profile of favipiravir and placebo were similar with the exception of transient elevation of uric acid that resolve with cessation of dosing.

In the randomised clinical trial comparing Favipiravir and Arbidol there was no difference in incidence of overall or organ specific adverse effects apart from a higher rate of elevated uric acid with favipiravir (13% vs 3%)

	≥ 1%	0.5 - < 1%	< 0.5%
Hypersensitivity		Rash	Eczema, pruritus
Hepatic	AST (GOT) increased, ALT (GPT) increased, γ-GTP increased		Blood ALP increased, blood bilirubin increased
Gastrointestinal	Diarrhoea (4.79%)	Nausea, vomiting, abdominal pain	Abdominal discomfort, duodenal ulcer, haematochezia, gastritis
Hematologic	Neutrophil count decreased, white blood cell count decreased		White blood cell count increased, reticulocyte count decreased, monocyte increased
Metabolic disorders	Blood uric acid increased (4.79%), blood triglycerides increased	Glucose urine present	Blood potassium decreased
Respiratory			Asthma, oropharyngeal pain, rhinitis, nasopharyngitis
Others			Blood CK (CPK) increased, blood urine present, tonsil polyp, pigmentation, dysgeusia, bruise, vision blurred, eye pain, vertigo, supraventricular extrasystoles

Table 2: Adverse reactions observed in Japanese clinical studied and global phase III clinical trials

16.2.8 TERATOGENIC EFFECTS

Genotoxicity studies indicate that favipiravir does not pose a clinical genotoxic risk, however, favipiravir was shown to be teratogenic in rats and rabbits., mice and monkeys. The no observed adverse effect level (NOAELs) for developmental effects were 100, 20, 300 and 100 mg/kg/day for mice, rats, rabbits and monkeys, respectively. Therefore favipiravir is contraindicated in pregnant females, those who may become pregnant, or those who are nursing.

Due to the effect on embryo-fetal development seen in animals, and the presence of favipiravir in semen for several days after the end of therapy, the recommendation is for male and female contraception for seven days following the end of treatment

A human testicular toxicity study was run to determine if there are effects on human spermatogenesis. No abnormality in testicular function tests was observed; however, this study was performed at doses less than those to be used in this study. Due to the effect on embryo-fetal development seen in animals, and the presence of favipiravir in semen for several days after the end of therapy, the recommendation is for male and female contraception for seven days following the end of treatment, in the dose regimen for influenza.

16.2.9 DRUG-DRUG INTERACTIONS

Favipiravir is not metabolised by cytochrome p-450 enzymes but is mostly metabolised by aldehyde oxidase (AO) and partly metabolised by xanthine oxidase (XO). Favipiravir partially inhibits AO and CYP2C8.

Therefore, Favipiravir should be administered with caution with

- Drugs known to significantly inhibit AO activity (e.g., pyrazinamide, amitriptyline, chlorpromazine, clomipramine, clozapine, erythromycin, ketoconazole, nortriptyline, quetiapine, raloxifene, perphenazine, promethazine, propafenone, tamoxifen, thioridazine).
- Drugs metabolized by the AO pathway (e.g., famciclovir, hydralazine, lamivudine, sulindac, zaleplon, ziprasidone).

Note the package insert for Favipiravir only recommends administering Favipiravir with care for the following medications: Pyrazinamide, Repaglinide, Theophylline, Famciclovir and Sulindac and provides the following advice.[7]

Drug	Signs, Symptoms, Treatment	Mechanism
Pyrazinamide	Blood uric acid level with pyrazinamide alone was 11.6 mg/dL but when co-administered with Favipiravir 1200 mg / 400 mg BD increased to 13.9 mg/dL	Increased reabsorption of uric acid in the renal tubule

Repaglinide	Blood levels of repaglinide may increase	Inhibition of CYP2C8
Theophylline	Blood levels of theophylline may increase	Interaction with xanthine oxidase may increase Favipiravir levels
Famciclovir	Famciclovir efficacy may be reduced	Inhibition of aldehyde oxidase by Favipiravir may decrease famciclovir levels
Sulindac	Sulindac efficacy may be reduced	Inhibition of aldehyde oxidase by Favipiravir may decrease sulindac levels

In addition, the medications Dexamethasons and Remdesivir are being considered standard of care for the treatment of COVID-19 in may centres. Importantly Favipiravir is not expected to have any drug-drug interactions or additional toxicities with co-administration with these agents.[10]

16.2.10 OTHER SAFETY CONSIDERATIONS

Due to the excellent safety profile of favipiravir the need for screening bloods (FBE, UEC, LFT) will be reviewed after the first 20 participants have completed study drug and follow-up safety bloods (Day 21 or 28 for community participants, and earlier for hospitalised participants). If the committee is satisfied that there is no significant signal of drug related laboratory adverse events, then a recommendation to proceed with community recruitment without the need for baseline laboratory testing can be considered. An example, of treatment emergent change in laboratory parameters that would lead to a recommendation to continue with baseline laboratory tests would be 2 or more drug related Grade 2 events such as increase in ALT or decrease in neutrophil count from baseline.

Transient uric acid elevations have been reported after receiving Favipiravir. With dosing for 5 days in influenza trials in 682 men and 971 women the pooled mean uric acid concentration rose from 0.35 to 0.45 mmol/L in men and from 0.26 to 0.42 mmol/L in women. None of these 1653 individuals experienced any clinically identifiable AE known to be associated with acute or chronic elevations of uric acid (e.g gout or renal stone formation). These individuals had a decline in their urate to normal levels over the following 10 days. [11]
Of note these elevations in uric acid are either still within the reference range for normal or just above the normal reference range (Male: 0.20-0.45 mmol/L., Female: 0.15-0.40 mmol/L).

In addition, multiple smaller trials have given higher or prolonged dosing of Favipiravir with no changes in safety profile or clinical events related to transient increases in serum uric acid. These include:
- The JP120 study where 8 Healthy individuals received 1800 mg twice daily on Day 1, followed by 800 mg twice daily from Day 2 to Day 21, and a single dose of 800 mg on Day 22. All eight subjects had blood uric acid increase, without clinical episodes of gout. Two people in this study had mild ALT and AST elevations. The LFT abnormalities and uric acid values trended towards normalization after study completion
- The US121 study that was a Phase 1 dose escalation trial in healthy subjects In this study the first cohort received Day 1: 1800mg twice daily and Days 2-10 800 mg twice daily. The second cohort received Day 1: 1800mg twice daily and Days 2-10 1000 mg twice daily. Adverse effects similar to that seen in the influenza studies with 5 days treatment (Day 1: 1800mg twice daily and Days 2-5 800 mg twice daily)

- A Japanese trial for Severe Fever with Thrombocytopenia Syndrome. Dosing was Day 1: 1800mg twice daily Days 2-10 800 mg twice daily. Patients tolerated this treatment well
- The JIKI Trial, an pen label clinical trial in Ebola patients in West Africa. Dosing was Day 1: 2400 mg, 2400 mg and 1200 mg on (given three times daily) then 1200 mg twice daily for up to nine days. 111 patients analysed and 60 died of uncontrolled Ebola. No Favipiravir discontinuations occurred due to concerns for toxicity. The investigators concluded Favipiravir was well tolerated and acceptable to study in higher doses.[11,12]

Based on this lack of clinical events in standard 5 day dosing and studies with prolonged dosing there will no uric acid monitoring in the VIRCO trial. Another consideration is that uric acid monitoring has the potential to unblind investigators to treatment allocation. As clinically relevant events have not been reported and the potential adverse impact on blinding this monitoring will not be performed
